# Supplementary material for: Polyketide synthase gene clusters in the ericoid mycorrhizal fungus Oidiodendron maius: genome-wide prediction, expression profiling, and functional characterization of knockout mutants
Source: Front Plant Sci. 2026 Sep 16;17:1924430. doi: 10.3389/fpls.2026.1924430 (PMC13623903; doi:10.3389/fpls.2026.1924430)
Supplement: Supplementary file 1 [file SupplementaryFile1.zip › Supplementary Materials/Supplementary Folder 1.zip!/Oidma1_AssemblyScaffolds_Repeatmasked/knownclusterblast/cluster7/jgi.p_Oidma1_107607_mibig_hits.html]

| MIBiG Protein | Description | MIBiG Cluster | MiBiG Product | % ID | % Coverage | BLAST Score | E-value |
| --- | --- | --- | --- | --- | --- | --- | --- |
| EPE34326.1 | heterokaryon\_incompatibility\_protein | BGC0001035 | Pneumocandin\_biosynthetic\_gene\_cluster | 46 | 63.358778626 | 87 | 9.1e-18 |
| EPE34353.1 | hypothetical\_protein | BGC0001035 | Pneumocandin\_biosynthetic\_gene\_cluster | 40 | 74.0458015267 | 72 | 4e-13 |
